# The effect of call libraries and acoustic filters on the identification of bat echolocation

**DOI:** 10.1002/ece3.1201

**Published:** 2014-08-22

**Authors:** Matthew J Clement, Kevin L Murray, Donald I Solick, Jeffrey C Gruver

**Affiliations:** 1 United States Geological Survey, Patuxent Wildlife Research CenterLaurel, Maryland, 20708; 2 Western EcoSystems Technology Inc.Bloomington, Indiana, 47404; 3 Western EcoSystems Technology Inc.Cheyenne, Wyoming, 82001

**Keywords:** Acoustic surveys, Anabat, Analook, bat detectors, Chiroptera, classification, cross-validation, discriminant function analysis, *Myotis sodalis*, species identification

## Abstract

Quantitative methods for species identification are commonly used in acoustic surveys for animals. While various identification models have been studied extensively, there has been little study of methods for selecting calls prior to modeling or methods for validating results after modeling. We obtained two call libraries with a combined 1556 pulse sequences from 11 North American bat species. We used four acoustic filters to automatically select and quantify bat calls from the combined library. For each filter, we trained a species identification model (a quadratic discriminant function analysis) and compared the classification ability of the models. In a separate analysis, we trained a classification model using just one call library. We then compared a conventional model assessment that used the training library against an alternative approach that used the second library. We found that filters differed in the share of known pulse sequences that were selected (68 to 96%), the share of non-bat noises that were excluded (37 to 100%), their measurement of various pulse parameters, and their overall correct classification rate (41% to 85%). Although the top two filters did not differ significantly in overall correct classification rate (85% and 83%), rates differed significantly for some bat species. In our assessment of call libraries, overall correct classification rates were significantly lower (15% to 23% lower) when tested on the second call library instead of the training library. Well-designed filters obviated the need for subjective and time-consuming manual selection of pulses. Accordingly, researchers should carefully design and test filters and include adequate descriptions in publications. Our results also indicate that it may not be possible to extend inferences about model accuracy beyond the training library. If so, the accuracy of acoustic-only surveys may be lower than commonly reported, which could affect ecological understanding or management decisions based on acoustic surveys.

## Introduction

Acoustic surveys are a common survey tool for a variety of vocal taxa. Historically, these surveys have relied on qualitative methods for species identification, but a variety of quantitative methods has been introduced in recent decades. Fully quantitative species identification should make surveys more objective, repeatable, and cost efficient (Digby et al. 2013). Accordingly, various quantitative models have been evaluated for surveys of birds (Acevedo et al. 2009), anurans (Waddle et al. 2009), cetaceans (Oswald et al. 2003), primates (Mielke and Zuberbühler 2013), bats (Armitage and Ober 2010), and other taxa. Although the biology of these species varies, acoustic identification methods are often quite similar. The identification process typically involves collecting a library of calls of known species identity, extracting time and frequency (and sometimes amplitude) data from call spectrograms, and using statistical techniques to predict the species identity of vocalizations recorded during acoustic surveys. However, research has focused primarily on the final step, statistical discrimination of calls (e.g., Preatoni et al. 2005; Acevedo et al. 2009). Methods include discriminant function analysis, neural networks, classification trees, and other techniques (Parsons and Jones 2000; Oswald et al. 2003; Trifa et al. 2008). However, the first two steps, collecting a library of known calls and extracting parameters from calls, provide the foundation for statistical models and therefore demand methodological rigor as well.

Call libraries represent a collection of data points sampled from a population of animal calls. As such, unbiased sampling is critical to developing valid statistical models. If the call library is not representative, the resulting statistical models will yield biased parameter estimates. Given that vocalizations are known to vary systematically in a variety of contexts, there is a risk of assembling unrepresentative call libraries. For example, anuran calls vary with geographic location (Snyder and Jameson 1965), bird calls can vary among flocks (Nowicki 1983), primate calls can differ among individuals (Price et al. 2009), cetacean calls may vary with environmental noise (Parks et al. 2007), and bat calls vary with habitat (Broders et al. 2004). If the sample of calls in a library represents a limited set of behaviors, habitats, and other relevant factors, then the library will not be representative and we expect that statistical models will be poorly suited to identifying animals recorded under different circumstances.

After compiling a library, calls must be quantified prior to statistical analysis. When not manually measured, call quantification can be accomplished using a “filter,” which is an algorithm that both selects sounds to analyze and measures sound parameters. Filters may be built into available sound analysis software packages, and they may be more or less customizable. Differences among filters may cause them to select different sounds, which is likely to affect the mean and standard deviation of call parameters. A poor filter may also select inappropriate call fragments or environmental sounds (e.g., rain and wind; Waddle et al. 2009). Such noise can be subjectively edited, but at the cost of time, objectivity and repeatability (Digby et al. 2013). In addition, filter settings can affect the measurement of a selected call. Given that filters affect the call parameters supplied to statistical identification models, they are likely to affect the outcome of those models.

Because bats are inaudible to humans, bat researchers are especially reliant on ultrasonic bat detectors to record bat calls, and various quantitative methods for species identification. These tools are used by academics, ecological consultants, and government agencies to assess bat behavior, habitat preferences, and presence or absence. In many cases, acoustic surveys for bats inform important wildlife management decisions. For example, acoustic surveys for bats are commonly used to assess the ecological risks at proposed wind farms (Pennsylvania Game Commission 2007; Ohio Department of Natural Resources 2009). In addition, the U.S. Fish and Wildlife Service (USFWS) is currently amending survey guidelines for the federally endangered Indiana bat *Myotis sodalis* to allow acoustic surveys instead of mist-net surveys (USFWS 2014). These presence/probable-absence determinations for Indiana bats have economic significance for proponents of development projects, as well as ecological significance for bat populations, so accurate identifications are important.

The important role of call libraries and call quantification in acoustic surveys for bats is sometimes acknowledged, but the actual effects have rarely been investigated. For example, the many studies quantifying the effect of geographic variation, local habitat, and bat behavior on echolocation characters typically acknowledge the potential for these factors to affect species identification (Thomas et al. 1987; Murray et al. 2001; Law et al. 2002; Berger-Tal et al. 2008). However, this potential problem has not been investigated empirically by, for example, testing a model developed in one region on calls from another region. Despite a recent suggestion that filter settings may be important to bat species identification (Britzke et al. 2013), little has been published other than a comparison of call parameters generated by a filter and manual measurement (Britzke and Murray 2000). Therefore, our goals were to 1) select published and unpublished filters and assess their impact on the selection of sounds, the measurement of bat call parameters, and results of quantitative species identification, and 2) examine how using an independent library of bat calls for model validation affects estimates of model accuracy.

## Materials and Methods

### Call libraries

The issues of sampling bat echolocation calls and filtering ultrasonic data apply to both full-spectrum and zero-crossing bat detectors (see Parsons and Szewczak 2009; for an introduction to bat detector types). In contrast to full-spectrum detectors, zero-crossing detectors do not record sound amplitude, resulting in less data being recorded, but also reducing data storage and computing costs. For this study, we used data from zero-crossing bat detectors because they are widely used in North America, we have access to a library of bat calls recorded with zero-crossing detectors, and the available software allows customization of filters. We refer to a single emission of ultrasonic sound as a pulse, and a series of pulses with interpulse intervals <1 s as a pulse sequence or sequence (Jones and Siemers 2011). We also use “call” as a general term for vocalizations, when the distinction between pulse and pulse sequence is not essential.

We obtained a library of echolocation calls of known species identity that had been previously recorded for use in other studies (e.g., Murray et al. 2001; Britzke et al. 2011). The 1556 pulse sequences were recorded by multiple researchers from 1997 to 2011 in 14 states in the eastern United States (Allen et al. 2010; Britzke et al. 2011). Calls were recorded with Anabat II, SD1, and SD2 ultrasonic frequency division bat detectors (Titley Electronics, Ballina, NSW, Australia). Calls were recorded by several methods, including from bats marked with chemiluminescent tags and bats exiting known roost sites (Britzke 2003). Recording was completed using active recording techniques (manually orienting the bat detector toward active bats) in open areas to maximize the length and quality of recordings (O'Farrell et al. 1999). Most widespread bat species in eastern North America were recorded, including big brown *Eptesicus fuscus*, silver-haired *Lasionycteris noctivagans*, eastern red *Lasiurus borealis*, hoary *L. cinereus*, gray *Myotis grisescens*, eastern small-footed *M. leibii*, little brown *M. lucifugus*, northern long-eared *M. septentrionalis*, Indiana *M. sodalis*, evening *Nycticeius humeralis*, and tri-colored bats *Perimyotis subflavus*. We used this set of pulse sequences, which we refer to as the main library, to assess how different filters select and measure bat pulses.

We compiled a second set of 13,801 sound files that consisted entirely of non-bat sounds recorded during passive acoustic surveys. We refer to this set of sounds as the non-bat library, and we used it to assess how well the filters exclude non-bat sounds.

The main library was collected in two phases, with 53% of calls collected prior to 2001 and 47% collected after 2001, with different personnel, equipment, and sites involved in each collection phase. Therefore, we treated the two collection phases as independently collected sub-libraries, although we included all eastern small-footed bat calls in both sub-libraries, due to a small sample size. We used these sets of calls, which we name the early library and the late library, to assess how different libraries affect estimates of call identification rates and to determine whether results can be generalized among libraries.

### Call filters

We filtered bat calls using program AnalookW (Titley Electronics, Ballina, NSW, Australia). A filter is a set of rules that instructs AnalookW to select sounds that meet filter criteria and then measure parameters from selected sounds. Our plan was to compare the performance of several filters from the literature, but we found a paucity of published filters. Most papers either cited the Britzke and Murray (2000) filter (e.g., Loeb and O'Keefe 2006; Duchamp and Swihart 2008; Hein et al. 2009), or they used an unspecified custom filter (e.g., O'Farrell et al. 2000; Rodhouse et al. 2011). In addition, the USFWS is currently evaluating the call analysis software, BCID (Bat Call Identification, Inc., Kansas City, MO) for possible inclusion in the revised summer survey guidelines for Indiana bats (U.S. Fish and Wildlife Service 2014). Therefore, we compared the Britzke and Murray (hereafter, BM) filter to the BCID filter (v. 10.0) and two filters we developed (WEST 1 and WEST 2) as part of our work surveying bat communities (Table1). The BM filter was developed in the DOS-based version of Analook (v. 4.7; Analook predates AnalookW) to select identifiable frequency-modulated pulse sequences (Britzke and Murray 2000). The BCID filter was designed to select identifiable pulse sequences for use in proprietary automated identification software (BCID, C.R. Allen, personal communication). The WEST 1 filter was designed to select pulse sequences for qualitative identification from data sets with little noise, while WEST 2 was designed for noisy data sets. Because BCID uses options available in AnalookW v. 3.7, while WEST 1 and WEST 2 use features available in AnalookW v. 3.8, it was necessary to use both versions of AnalookW in our analysis. Although we used the BCID filter, we did not use the BCID classification algorithm, and therefore, our results are presumably different from results that would be obtained with BCID software.

**Table 1:** Pulse selection rules used by four Analook filters

Parameters	BM	BCID	WEST 1	WEST 2
Smoothness (%)	15	12	15	10
High Start (T/F)	F	F	T	T
Max Change (kHz)			+2, −4	+2, −4
Ignore Fragments (µs)		2200		
Join Fragments (µs)		2000		
Reject both calls with gap (ms)			2	2
Body Over (µs)	240	2400	1000	2000
Fc (kHz)			15–60	15–60
Fmax (kHz)		17–120		
Fmin (kHz)		16–60		
Sc (octaves/s)			−100–1000	−100–1000
Sweep (kHz)	6–300	3–70	0.1–60	0.1–60
S1(octaves/s)			−30–9999	30–9999
Duration (ms)	1–30	1–20	1–30	2–30
Min Number of Calls (number)	5	5	5	5
Time for Calls (s)	5	15	5	5
Min Time Between Calls (ms)			50	50
PMC (%)				8–9999
Synthetic Line 1				
Min			0	0
Max			60	60
X Variable			Dur	Dur
X1			6	6
X2			0	0
Y Variable			Sweep	Sweep
Y1			0	0
Y2			3.5	3.5
Synthetic Line 2				
Min			0	0
Max			1000	1000
X Variable			Dur	Dur
X1			3.25	3.25
X2			1.75	1.75
Y Variable			Sweep	Sweep
Y1			0	0
Y2			8	8

A similar analysis performed with full-spectrum detectors would differ primarily in the details. For example, different software would be required to select and measure pulses (e.g., Sonobat; Corcoran 2007). Also, because full-spectrum detectors record amplitude data, additional filter rules could be created based on sound amplitude (Corcoran 2007). However, the basic goal of establishing rules for selecting and measuring pulses would not change.

### Analysis

While filters can help automate acoustic analysis, they select any recorded sounds that meet their criteria, including pulse fragments or noise produced by non-bat sources. Therefore, it is common to manually edit sound files after filtering to remove additional noise or low-quality pulses (e.g., Britzke 2003; Loeb and O'Keefe 2006; Duchamp and Swihart 2008; Gorresen et al. 2008; Armitage and Ober 2010). One motivation for manual review is to remove noise that would otherwise incorrectly be included in either a training or validation data set. A second issue is human observers may be better able to detect faint or low-quality calls (Digby et al. 2013).

However, manually selecting individual pulses for inclusion in a data set is a subjective process that introduces variability into both the selection of pulses (Britzke and Murray 2000; Fritsch and Bruckner 2014) and the measurement of pulse parameters (Britzke and Murray 2000). Such inconsistency reduces the repeatability of the work (Barclay 1999; Fritsch and Bruckner 2014) and makes it difficult to compare results from different studies. Furthermore, it increases the time required to complete the analysis (Armitage and Ober 2010; Digby et al. 2013). Of particular concern is the potential for selection bias when manually selecting pulses for analysis (Berk 1983). If distinctive pulses are more likely to be manually selected, the sample will not be representative and model error rates will likely be underestimated. The same issues apply to full-spectrum recordings because such data are also typically manually reviewed after filtering (Walters et al. 2012; Fritsch and Bruckner 2014). To minimize these problems, we did not manually edit sounds and accepted the output of each filter *in toto*.

We assessed the effect of filters on call selection by comparing the number of pulses and pulse sequences selected by each filter when applied to the main library. We also identified a consensus set of pulses and pulse sequences that were selected by all four filters. We assessed the ability of filters to exclude non-bat sounds by comparing the number of pulses and pulse sequences selected from the non-bat library by each filter.

We assessed the effect of filters on pulse parameter measurement by calculating the mean and standard deviation of pulse parameters selected by each filter. Examination of Q-Q plots indicated parameter values were not normally distributed, so we used a Kruskal–Wallis test (*α* = 0.05) and pairwise Wilcoxon rank sum tests (*α* = 0.05) with a Holm multiple-comparison adjustment (Holm 1979) to determine significant differences among means. Differences in mean parameter values could be due to the different pulses selected or different measurement of the same pulses. Therefore, we also examined the consensus set of pulses, which were selected by all filters. We applied the four filters to the consensus pulses to obtain new pulse parameter measurements and repeated the Kruskal–Wallis and pairwise Wilcoxon rank sum tests.

Next, we evaluated the effect of filters on quantitative identification of pulse sequences. Our general approach was to apply the four filters to the main library to generate four pulse parameter data sets, develop a classification model from each data set, and then use cross-validation to measure the performance of the four models. We used the same covariates in all four models so that the only difference among analyses was the filter used.

We used discriminant function analysis (DFA) for our classification model because it is most commonly used to quantitatively identify bat calls (Biscardi et al. 2004), although other statistical methods may be appropriate as well (Preatoni et al. 2005). A DFA generates a set of uncorrelated combinations of continuous predictor variables, called canonical discriminant functions, that maximally separate the observations into groups (Venables and Ripley 2002). In this case, the predictor variables are pulse parameters extracted by AnalookW and the groups are individual bat species.

**Figure 1:**
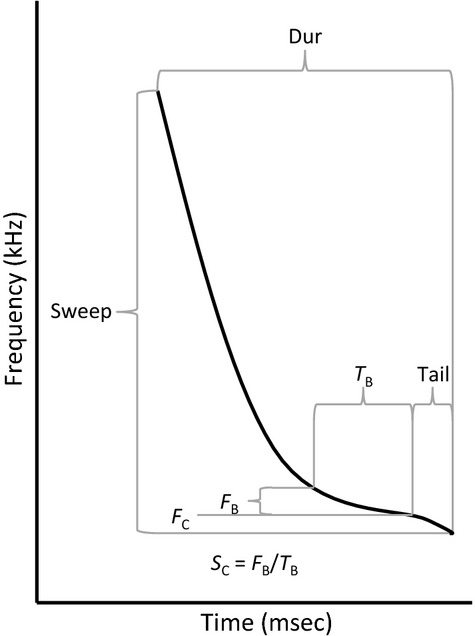
Schematic of a bat echolocation pulse and relevant parameters. Dur = pulse duration (ms), Sweep = total pulse bandwidth (kHz), T_B_ = duration (s) of the body (the flattest portion of the call), F_B_ = bandwidth (octaves) of the body, Sc = slope of the body (F_B_/T_B_; octaves/s), Fc = frequency at the end of the body (kHz), and Tail = duration (ms) of the pulse after the body.

To facilitate comparison of the filters, we included the same covariates in all four DFAs. We included all uncorrelated (Pearson *R*^2^ < 0.5) pulse parameters in the models as covariates. When two parameters were correlated, we selected the one that was correlated with fewer parameters, or has been reported as more reliable or diagnostic (Corben 2004; Britzke et al. 2011). We considered 15 pulse parameters provided by AnalookW and two derived parameters. The five selected parameters included pulse duration (Dur), frequency at the end of the body (Fc), slope of the body (Sc), total pulse bandwidth (Sweep), and duration of the pulse after the body (Tail) where the body is defined as the flattest portion of the pulse (Fig.1). Note that although each DFA used the same covariates, each DFA analyzed different data because the data sets were generated by different filters.

Given that the four pulse parameter data sets failed a Shapiro–Wilk test for multivariate normality (*P* < 0.001) and a Box's M test for homogeneity of the variance–covariance matrix (*P* < 0.001), we used a quadratic DFA (qDFA; Venables and Ripley 2002). We completed qDFAs in Program R (version 2.15.1, R Development Core Team 2013) using the “qda” function in package MASS (Venables and Ripley 2002). The models included the five uncorrelated variables, and the prior probabilities of group membership were equalized. The qDFA classified individual pulses to species based on the highest posterior probability. We then assigned a pulse sequence to a species if the majority of pulses (all sequences included at least five pulses; Table1) could be assigned to one species. If no species was associated with a majority of pulses, we classified the pulse sequence as “unknown.”

We quantified qDFA performance on the main library using leave-one-out cross-validation (Fielding and Bell 1997). In this procedure, one pulse sequence is removed from the data set, a qDFA is fit to the remaining sequences, and then, the resulting model is used to predict the identity of the withheld sequence. The leave-one-out procedure is repeated for every pulse sequence in the data set and the percentage of sequences that were correctly classified, incorrectly classified, or classified as unknown is recorded. Specifically, if there were *N*_*i*_ known pulse sequences of species *i* in the library, and *n*_*i*_ were correctly identified as species *i*, *n*_*j*_ were incorrectly identified as other species, and *N*_*i*_ − *n*_*i*_ − *n*_*j*_ were classified as unknown, then *n*_*i*_/*N*_*i*_ was the correct classification rate, *n*_*j*_/*N*_*i*_ was the incorrect classification rate, and (*N*_*i*_ − *n*_*i*_ − *n*_*j*_)/*N*_*i*_ was the unknown rate. To obtain overall classification rates, we used an un-weighted average of the rates for the 11 species, 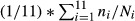. We then used a bootstrap procedure to estimate 90% confidence intervals around cross-validation estimates (Fielding and Bell 1997). In this procedure, a new data set of pulse sequences, equal in size to the original data set, is generated by sampling with replacement from the original data set. The qDFA and cross-validation are repeated on the new data set. We repeated this bootstrap 1000 times and used the 5th and 95th quantiles of the estimated classification rates as a 90% confidence interval. If confidence intervals did not overlap, we considered the differences to be statistically significant.

Finally, we examined how using independent data for model validation affects estimates of model accuracy. The typical approach to model validation is to divide a call library into training and validation data, using leave-one-out cross-validation, k-fold partitioning, or a similar approach (e.g., Biscardi et al. 2004; Berger-Tal et al. 2008; Britzke et al. 2011). However, any artifacts of nonrandom sampling in the training data set are likely to be in the validation data set, which can inflate estimates of correct classification rates (Chatfield 1995). A more robust approach is to collect independent data to test the model (Pearce and Ferrier 2000). Independent data may reveal the poor predictive ability of a model and the unreliability of correct classification rates obtained from internal cross-validation (Morrison et al. 1987; Fielding and Haworth 1995). Therefore, we used the early and late libraries as independent call libraries. First, we filtered the early library with the WEST 2 filter, performed a qDFA with the five pulse parameters described previously and assessed it using internal cross-validation. In this case, we used the same leave-one-out cross-validation and bootstrapping as before, so that the early library was used to both fit and validate the model. We then assessed the same model with external validation. In this case, we used the entire early library to fit the model and the entire late library to validate the model. We bootstrapped the data 1000 times to estimate 90% confidence intervals. We compared the correct classification rates of the internal and external validation, with non-overlapping confidence intervals considered to be significant. We then repeated this process, reversing the early and late libraries.

## Results

Of 1556 bat pulse sequences in the main library, the BCID filter selected the most sequences and the BM filter selected the most pulses, while WEST 2 selected the fewest of each (Table2). Although the BCID and BM filters selected more total pulse sequences, the effect varied across species, with WEST 1 selecting more sequences for some low bandwidth species such as hoary bats, silver-haired bats, and tri-colored bats. When we applied the filters to the 13,801 files in the non-bat library, all filters erroneously selected non-bat sounds, with BM selecting by far the most, and WEST 2 selecting the fewest (Table2).

**Table 2:** Echolocation pulse sequences and pulses, as well as non-bat noises, selected by AnalookW filters, by species^1^. The consensus filter is a composite filter that selects only calls and pulses selected by all four filters

	Filter	Total Bat	EPFU	LABO	LACI	LANO	MYGR	MYLE	MYLU	MYSE	MYSO	NYHU	PESU	Noise
Sequences	BM	1456	404	65	53	68	79	12	162	84	231	100	198	8677
	BCID	1493	404	67	76	87	79	9	162	71	229	101	208	172
	W1	1339	283	62	90	80	73	11	147	81	223	87	202	53
	W2	1060	222	54	67	64	71	5	123	51	166	66	171	1
	Consensus	992	221	53	37	48	71	5	122	49	165	62	159	1
Pulses	BM	61,617	21,478	2080	1042	1530	3213	385	7246	3426	11,188	3871	6158	379,458
	BCID	47,788	16,398	2118	1583	2109	2823	154	5640	1220	5524	3669	6550	3402
	W1	25,850	4096	1382	1577	2234	1562	203	3146	1615	4586	1607	3842	574
	W2	16,968	2886	1046	1071	1466	1320	75	2346	623	2466	971	2698	23
	Consensus	13,459	2427	858	344	732	1212	62	2222	532	2102	812	2156	23

1 EPFU, *Eptesicus fuscus*; LABO, *Lasiurus borealis;* LACI, *L. cinereus;* LANO, *Lasionycteris noctivagans;* MYGR, *Myotis grisescens;* MYLE, *M. leibii;* MYLU, *M. lucifugus;* MYSE, *M. septentrionalis;* MYSO, *M. sodalis;* NYHU, *Nycticeius humeralis;* PESU, *Perimyotis subflavus*.

Mean pulse parameters differed among the filters. Considering Indiana bat pulse sequences from the main library, parameter measurements differed significantly among BM, BCID, and WEST 1 for all five parameters, while WEST 2 differed for three parameters (Table3). Mean Fc differed by 2.2 kHz and mean Sc differed by 32 octaves/s among filters. In comparison, Indiana bats recorded in different regions differ by 1.4 kHz and 25 octaves/s (Murray et al. 2001). Results for the other ten bat species under consideration showed a mix of larger and smaller differences among filters (Tables S1–S10).

**Table 3:** Means of *Myotis sodalis* pulse parameters selected from the main library by different AnalookW filters and means of a consensus set of pulses selected by all four filters. Standard deviations given in parentheses. Parameters explained in text. Different letters indicate significantly different means (*α* = 0.05) according to pairwise Wilcoxon rank sum tests.

Parameters	All selected				Consensus			
	
	BM (*n* = 11,188)	BCID (*n* = 5524)	WEST 1 (*n* = 4586)	WEST 2 (*n* = 2466)	BM (*n* = 2102)	BCID (*n* = 2102)	WEST 1 (*n* = 2102)	WEST 2 (*n* = 2102)
Dur (ms)	2.4 a (1.0)	3.2 b (0.7)	2.8 c (0.9)	3.1 d (0.7)	3.2 b (0.7)	3.2 b (0.6)	3.2 b (0.7)	3.2 b (0.6)
Sweep (kHz)	19.6 a (10.7)	25.2 b (10.2)	22.1 c (9.8)	23.5 d (9.0)	24.9 be (9.1)	24.8 be (9.0)	24.8 be (8.9)	24.5 de (8.8)
Fc (kHz)	45.6 a (8.0)	44.0 b (3.6)	43.4 c (2.8)	44.0 b (2.8)	45.6 a (4.0)	44.2 b (3.4)	42.9 d (2.6)	43.8 b (2.7)
Sc (octaves/s)	185.3 a (171.0)	152.9 bc (44.0)	161.5 d (54.1)	160.4 cde (42.0)	144.6 f (35.1)	151.3 bef (38.7)	144.7 bf (39.9)	158.2 cde (40.8)
Tail (ms)	0.7 a (0.4)	0.5 b (0.5)	0.2 c (0.3)	0.4 d (0.3)	0.8 e (0.5)	0.5 b (0.5)	0.3 c (0.4)	0.4 d (0.3)

When we limited parameter measurement to the consensus pulses, the effect varied by parameter. For three parameters associated with the Body (Fc, Sc, and Tail), most pulse parameters were still significantly different, indicating the differences were primarily due to how filters measure parameters (Table3). For two parameters unrelated to the Body (Dur, Sweep), differences among filters were no longer significant, indicating that the differences were primarily due to pulse selection and not parameter measurement. Results were similar for the other bat species analyzed (Tables S1–S10).

**Figure 2:**
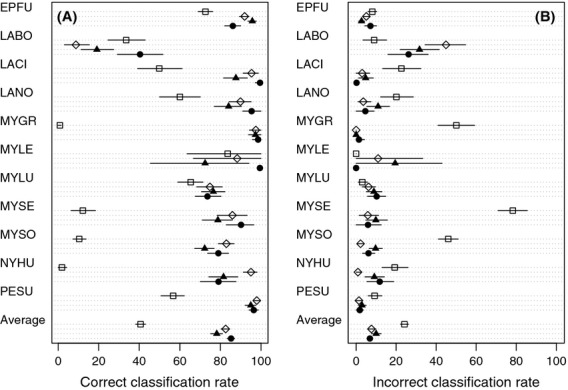
Results of a discriminant function analyses using different AnalookW filters (open square: Britzke-Murray, open diamond: BCID, filled triangle: WEST 1, filled circle: WEST 2). (A) Percent of bat calls selected by each filter that were correctly classified, with bootstrapped 90% confidence intervals. (B) Percent of bat calls selected by each filter that were incorrectly classified, with bootstrapped 90% confidence intervals. Remaining bat calls were classified as “unknown.” Species codes given in Table2.

The accuracy of qDFA predictions varied, depending on the filter used (Fig.2). Overall, the qDFA based on the WEST 2 filter had the highest percentage of correctly identified pulse sequences, followed by the BCID filter, WEST 1 filter, and BM filter, although confidence intervals for BCID overlapped WEST 1 and WEST 2. The qDFA based on the WEST 2 filter yielded the highest correct classification rate for six species, while BCID was highest for three species and WEST 1 was highest for two species. Results were similar for incorrect classifications, with WEST 2 yielding a lower, but not significantly different, rate than BCID and WEST 1, and the BM filter producing a significantly higher error rate.

**Figure 3:**
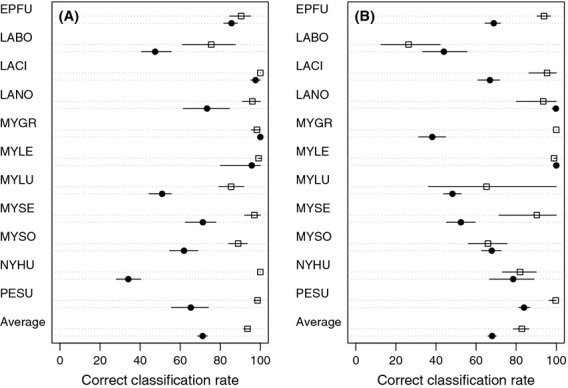
Results of a discriminant function analysis using the WEST 2 filter and different validation data sets (open square: training data used for model validation, closed circle: validation data independent of training data). (A) Early library, collected between 1999 and 2002, used for model training. (B) Late library, collected between 2005 and 2011, used for model training. Values are percent of bat calls correctly identified, with bootstrapped 90% confidence interval. Species codes given in Table2.

Overall correct classification rates in the early library were significantly lower when estimated using external validation (71%) instead of internal cross-validation (94%; Fig.3). The estimated correct classification rate was significantly lower for seven of the 11 species considered. For example, the correct classification rate for evening bats was estimated as 100% with internal cross-validation, but 34% with external validation. We found the same pattern when we examined the late library, with significantly lower estimates of correct classification rates using external validation (68%) rather than internal cross-validation (83%; Fig.3).

## Discussion

We found that filters affect all stages of echolocation identification, from selection of pulses and pulse sequences, to measurement of parameters, to results of a classification model. That different filters select different pulses and pulse sequences is not a surprising result, but it has important implications. First, it means that the choice of a filter can affect the content of a data set and the outcome of analysis. Second, if filters select many pulse fragments or non-bat sounds, a classification model will be trained on noise, rather than true bat calls. Accordingly, we would not recommend using the BM filter without manual review because it admitted high levels of noise. However, the WEST2 filter was nearly perfect at eliminating noise (Table2), demonstrating that filters without manual review are feasible.

We found that filters affect the measurement of pulses through both pulse selection and pulse measurement. It may be self-evident that different filters will select different pulses. However, the effect of filter settings on pulse measurement may not be fully appreciated. In particular, AnalookW defines the “body” as the flattest portion of an echolocation pulse and measures several characteristics of the body (Corben 2004). Two of these, frequency at the end of the body (Fc) and slope of the body (Sc), are typically among the most important predictors of bat species identity (Parsons and Jones 2000; Corben 2004; Armitage and Ober 2010; Britzke et al. 2011). However, the time increment over which slope is measured affects the size, shape, and location of the body, and therefore Fc and Sc as well. In AnalookW, this increment is controlled by the Body Over setting, but the same principle applies to other software that estimates a body and related parameters, including software designed for analyzing full-spectrum data (e.g., Sonobat; Corcoran 2007). Therefore, Body Over settings likely played a large role in the different pulse measurements we report. Furthermore, the effect of filters on some parameters was similar to the previously reported effect of geographic regions (Murray et al. 2001), reinforcing the importance of filters to acoustic analysis.

We found that filter settings had the potential to significantly affect qDFA results, although different filters can produce similar results. Specifically, results obtained with the BM filter were significantly worse than the other filters. We attribute this to the BM filter having been developed in an earlier version of Analook, which had fewer filtering capabilities. As a result, the BM filter selected some non-bat sounds that probably impaired its ability to distinguish among bat species. Overall correct classification rates from the other three filters were similar, suggesting that classifications are moderately robust to differences among filters. While the WEST 2 filter produced the highest correct classifications in this study, it also selected the fewest pulse sequences, which could result in more false negatives during surveys. Furthermore, the relative performance could change under different conditions, such as a different set of bat species, or a different statistical method for classification. However, we expect that the ability of the WEST 2 filter to exclude noise could lead to better performance on passively recorded bat calls.

Although we completed our analysis with no manual review of the filter output, there remains the question of whether an automated system or a manual system is preferable. In this study, all filters allowed some noise, and theoretically, a human observer could remove this noise. However, previous research shows that human observers do not consistently demarcate noise (Britzke and Murray 2000; Fritsch and Bruckner 2014), suggesting that manual review is not a simple solution to the issue of excluding noise. Human observers might be more sensitive at detecting weak, fragmented, or low-quality pulses (Corcoran 2007; Digby et al. 2013). However, such low quality pulses may be of little value for species identification (Jennings et al. 2008). Although filters in this study may have incorrectly included or excluded some sounds, correct classification rates for the better filters were reasonably robust to differences in the sounds selected (Table3). Furthermore, correct classification rates were similar to those reported in previous studies (e.g., Preatoni et al. 2005; Corcoran 2007; Armitage and Ober 2010; Britzke et al. 2011; Walters et al. 2012), suggesting that automated filters can compete against expert observers. Most importantly, we argue that the ultimate goal of a classification model is not to accurately classify known calls, but to classify unknown calls. If we mistakenly conflate these two goals, we may be tempted to select pulses from a library that maximize classification of known calls, but reduce classification of unknown calls (Berk 1983; Morrison et al. 1987; Fielding and Haworth 1995). If we use objective filters instead of subjective techniques, we can reduce the temptation and better achieve our goal of classifying unknown calls.

We also found that the composition of the call library significantly affected estimates of correct classification rates. Previous work has shown that bat calls collected under different circumstances have different characteristics. For example, pulse parameters may differ due to bat calls being collected in different regions (Murray et al. 2001), at different distances from obstacles (Broders et al. 2004; Siemers and Kerth 2006), while bats engage in different behaviors (Berger-Tal et al. 2008), or under different recording protocols (Parsons and Szewczak 2009). While these studies showed that recording circumstances affected pulse characteristics, our results show that these differences significantly affect classification. In particular, external validation indicated much lower correct classification rates than internal cross-validation.

Testing model classification ability with independent data is recommended as a more rigorous approach than cross-validation (Chatfield 1995; Pearce and Ferrier 2000). When we compared these two approaches, correct classification rates were significantly lower when we used external rather than internal cross-validation (Fig.3). If this pattern is common, then reported classification rates based on internal validation may be too optimistic. Several other ecological studies have also found that model accuracy decreased when applied to independent data. For example, an evaluation of models of bird abundance found prediction errors of 25–75% when tested with independent data (Morrison et al. 1987). A study of classification models designed to predict bird habitat also showed that prediction success was highly variable when models were applied to independent data (Fielding and Haworth 1995). An attempt to identify individual bats from their echolocation calls found that classification performance dropped dramatically when independent validation data were used (Siemers and Kerth 2006).

The lower classification rates estimated with independent validation data arise when the training data are not representative of the target population (Fielding and Haworth 1995). In the current study, both the training and validation data were recorded with active techniques (i.e., detectors were continually monitored and oriented toward active bats) in open areas. However, in many applications, the population of interest will include passively recorded bat calls in various habitats (e.g., Loeb and O'Keefe 2006). In addition, surveys are often conducted with weather-proofing devices, while library calls may be recorded without them. Given that habitat (Siemers and Kerth 2006), active recording techniques (O'Farrell et al. 1999), and weather-proofing (Britzke et al. 2010) are known to affect pulse characteristics, external validation with passively recorded pulse sequences may generate even lower correct classification rates than we report.

Efforts to compile libraries and filter calls for other taxa likely share some challenges with bats, but differ in other ways. The basic approach of comparing survey data to known calls is similar among taxa, as are many of the statistical methods used (e.g., Oswald et al. 2003). Furthermore, the problem of assuring a library is representative when vocalizations are affected by many covariates exists for other taxa as well. These similarities suggest that identification of other taxa will also be sensitive to the filter and library used. However, acoustic surveys for other taxa more commonly focus on territorial or mating calls, which are intended to communicate the species of the caller and therefore are stereotyped. In contrast, the primary function of bat echolocation is to enable foraging and orientation in darkness (Barclay 1999). Accordingly, bat echolocation is relatively plastic and bat species occupying similar foraging niches often produce similar calls (Siemers et al. 2001). Therefore, surveys of territorial or mating calls may be less sensitive to the filter and library used, relative to surveys of orientation calls, alarm calls, or other less distinct calls.

### Recommendations

Given that filters affect pulse selection, pulse measurement, correct classification rates, and model generality, filter design should be treated as an integral part of the acoustic identification process. Filters are often described as quantitative, objective, and repeatable, but we believe there is room for improvement (Fritsch and Bruckner 2014). First, filter descriptions in publications should be adequate to assist others in recreating them, which is not the current practice. Second, researchers should strive to eliminate manual filtering, as we have performed here. Although we used single filters, we can imagine cases where several filters would be used in a series to isolate bat calls. If manual filtering is the only practical approach, then the filtering criteria should be written in an explicit, objective, and repeatable manner. Finally, the effect of filters on results should be acknowledged. For example, a filter that seeks frequency-modulated pulses may exclude a disproportionate share of species with less frequency-modulated pulses (Table2). In a study of community composition, this type of bias could affect results and conclusions.

We found that validating with independent data yielded much lower estimates of correct classification rates. Therefore, when identifying bat calls, it may not be possible to extend inference beyond the training data set. Such limited inference has important implications. For example, acoustic-only surveys, such as those used for endangered Indiana bats (U.S. Fish and Wildlife Service 2014) may produce inaccurate estimates of the probability of presence. Fortunately, alternative approaches, such as newly developed false-positive occupancy models (Miller et al. 2011) can provide unbiased occupancy estimates even when identification errors occur, if acoustic surveys can feasibly be combined with capture surveys (Clement et al. 2014).

## Supporting Information

**Table S1.** Means of *Eptesicus fuscus* pulse parameters selected from the main library by different AnalookW filters and means of a consensus set of pulses selected by all four filters. **Table S2.** Means of *Lasiurus borealis* pulse parameters selected from the main library by different AnalookW filters and means of a consensus set of pulses selected by all four filters. **Table S3.** Means of *Lasiurus cinereus* pulse parameters selected from the main library by different AnalookW filters and means of a consensus set of pulses selected by all four filters. **Table S4.** Means of *Lasionycteris noctivagans* pulse parameters selected from the main library by different AnalookW filters and means of a consensus set of pulses selected by all four filters. **Table S5.** Means of *Myotis grisescens* pulse parameters selected from the main library by different AnalookW filters and means of a consensus set of pulses selected by all four filters. **Table S6.** Means of *Myotis leibii* pulse parameters selected from the main library by different AnalookW filters and means of a consensus set of pulses selected by all four filters. **Table S7.** Means of *Myotis lucifugus* pulse parameters selected from the main library by different AnalookW filters and means of a consensus set of pulses selected by all four filters. **Table S8.** Means of *Myotis septentrionalis* pulse parameters selected from the main library by different AnalookW filters and means of a consensus set of pulses selected by all four filters. **Table S9.** Means of *Nycticeius humeralis* pulse parameters selected from the main library by different AnalookW filters and means of a consensus set of pulses selected by all four filters. **Table S10.** Means of *Perimyotis subflavus* pulse parameters selected from the main library by different AnalookW filters and means of a consensus set of pulses selected by all four filters.
