# Supplementary material for: The effect of call libraries and acoustic filters on the identification of bat echolocation
Source: Ecol Evol. 2014 Aug 22;4(17):3482–93. doi: 10.1002/ece3.1201 (PMC4228621; doi:10.1002/ece3.1201)
Supplement: Supplementary file 1 [file ece30004-3482-sd1.docx]

**Table S1.** Means of *Eptesicus fuscus* pulse parameters selected from the main library by different AnalookW filters and means of a consensus set of pulses selected by all four filters. Standard deviations given in parentheses. Parameters explained in text. Different letters indicate significantly different means (α = 0.05) according to pairwise Wilcoxon rank sum tests.

|  |  | All Selected | | | |  | Consensus | | | |
| --- | --- | --- | --- | --- | --- | --- | --- | --- | --- | --- |
| Parameter | Units | BM  (n = 21,478) | BCID  (n=16,398) | WEST 1  (n = 4,096) | WEST 2  (n = 2,886) |  | BM  (n = 2,427) | BCID  (n = 2,427) | WEST 1  (n = 2,427) | WEST 2  (n = 2,427) |
| Dur  Sweep  Fc  Sc  Tail | Ms  kHz  kHz  octaves/s  ms | 3.8 a  (2.3)  14.6 a  (7.7)  32.5 a  (8.2)  156.4 a  (148.8)  0.9 a  (0.6) | 4.5 b  (2.1)  14.4 b  (8.6)  30.6 b  (4.4)  95.6 b  (50.7)  0.5 b  (0.6) | 5.7 c  (2.5)  17.8 c  (9.4)  29.5 c  (3.3)  87.8 c  (56.4)  0.5 c  (0.5) | 6.0 d  (2.5)  17.5 c  (9.6)  28.8 d  (2.7)  77.2 d  (42.8)  0.4 d  (0.5) |  | 6.3 e  (2.5)  19.1 d  (9.0)  29.5 e  (3.0)  79.8 d  (52.4)  0.9 e  (0.6) | 6.3 e  (2.4)  19.1 d  (9.0)  29.0 d  (2.8)  78.3 d  (43.6)  0.4 f  (0.5) | 6.3 e  (2.4)  19.1 d  (9.0)  28.9 d  (2.7)  78.6 d  (44.4)  0.4 f  (0.5) | 6.3 e  (2.5)  19.0 d  (9.0)  28.8 d  (2.5)  77.4 d  (4.1)  0.4 d  (0.5) |

**Table S2.** Means of *Lasiurus borealis* pulse parameters selected from the main library by different AnalookW filters and means of a consensus set of pulses selected by all four filters. Standard deviations given in parentheses. Parameters explained in text. Different letters indicate significantly different means (α = 0.05) according to pairwise Wilcoxon rank sum tests.

|  |  | All Selected | | | |  | Consensus | | | |
| --- | --- | --- | --- | --- | --- | --- | --- | --- | --- | --- |
| Parameter | Units | BM  (n = 2,080) | BCID  (n = 2,118) | WEST 1  (n = 1,382) | WEST 2  (n = 1,046) |  | BM  (n = 858) | BCID  (n = 858) | WEST 1  (n = 858) | WEST 2  (n = 858) |
| Dur  Sweep  Fc  Sc  Tail | Ms  kHz  kHz  octaves/s  ms | 5.7 a  (2.9)  17.2 a  (9.0)  39.7 a  (7.7)  90.0 a  (151.3)  0.9 a  (0.8) | 6.4 b  (2.6)  14.9 b  (9.5)  38.3 bc  (3.5)  39.5 b  (38.9)  0.5 bc  (0.6) | 6.7 b  (2.7)  15.3 b  (9.2)  38.2 bc  (3.8)  43.4 b  (50.9)  0.6 bc  (0.7) | 6.7 b  (2.4)  14.8 b  (8.7)  37.9 b  (3.6)  40.4 bc  (33.4)  0.6 c  (0.7) |  | 6.7 b  (2.3)  16.7 a  (8.2)  38.5 c  (3.4)  40.7 c  (30.9)  0.8 a  (0.7) | 6.6 b  (2.3)  16.7 a  (8.2)  38.3 bc  (3.4)  37.7 b  (30.1)  0.4 d  (0.6) | 6.7 b  (2.3)  16.7 a  (8.2)  38.3 bc  (3.4)  39.1 bc  (29.5)  0.5 bd  (0.6) | 6.6 b  (2.3)  16.6 a  (8.2)  38.3 bc  (3.4)  41.4 c  (30.7)  0.6 bc  (0.6) |

**Table S3.** Means of *Lasiurus cinereus* pulse parameters selected from the main library by different AnalookW filters and means of a consensus set of pulses selected by all four filters. Standard deviations given in parentheses. Parameters explained in text. Different letters indicate significantly different means (α = 0.05) according to pairwise Wilcoxon rank sum tests.

|  |  | All Selected | | | |  | Consensus | | | |
| --- | --- | --- | --- | --- | --- | --- | --- | --- | --- | --- |
| Parameter | Units | BM  (n = 1,042) | BCID  (n = 1,583) | WEST 1  (n = 1,577) | WEST 2  (n = 1,071) |  | BM  (n = 344) | BCID  (n = 344) | WEST 1  (n = 344) | WEST 2  (n = 344) |
| Dur  Sweep  Fc  Sc  Tail | Ms  kHz  kHz  octaves/s  ms | 8.6 a  (4.5)  12.0 a  (5.3)  23.5 a  (5.1)  77.1 a  (194.7)  1.4 a  (1.2) | 9.7 b  (4.0)  8.9 b  (5.5)  22.4 b  (2.9)  32.1 b  (33.0)  0.7 b  (0.8) | 10.6 c  (3.9)  6.8 c  (5.8)  21.3 c  (3.3)  25.0 c  (32.5)  2.1 c  (2.3) | 10.3 c  (3.8)  8.1 d  (5.4)  22.1 d  (2.7)  26.5 d  (22.3)  0.7 b  (0.8) |  | 11.3 d  (3.7)  10.4 e  (4.2)  23.4 a  (2.4)  26.2 b  (16.3)  1.8 c  (1.4) | 11.3 d  (3.7)  10.4 e  (4.2)  23.0 a  (2.2)  25.3 bd  (16.3)  0.9 d  (0.8) | 11.3 d  (3.7)  10.5 e  (4.2)  23.1 a  (2.2)  25.7 bd  (16.8)  1.2 a  (1.0) | 11.3 d  (3.7)  10.4 e  (4.1)  23.0 a  (2.2)  25.9 bd  (16.3)  0.9 d  (0.8) |

**Table S4.** Means of *Lasionycteris noctivagans* pulse parameters selected from the main library by different AnalookW filters and means of a consensus set of pulses selected by all four filters. Standard deviations given in parentheses. Parameters explained in text. Different letters indicate significantly different means (α = 0.05) according to pairwise Wilcoxon rank sum tests.

|  |  | All Selected | | | |  | Consensus | | | |
| --- | --- | --- | --- | --- | --- | --- | --- | --- | --- | --- |
| Parameter | Units | BM  (n = 1,530) | BCID  (n = 2,109) | WEST 1  (n = 2,234) | WEST 2  (n = 1,466) |  | BM  (n = 732) | BCID  (n = 732) | WEST 1  (n = 732) | WEST 2  (n = 732) |
| Dur  Sweep  Fc  Sc  Tail | Ms  kHz  kHz  octaves/s  ms | 7.6 a  (4.0)  11.7 a  (7.6)  27.3 a  (4.6)  95.8 a  (180.0)  1.1 a  (0.8) | 9.3 b  (3.3)  8.5 b  (4.8)  26.8 b  (1.3)  29.6 b  (41.7)  0.5 b  (0.6) | 11.0 c  (3.4)  6.4 c  (5.1)  26.3 c  (1.1)  23.1 c  (61.8)  0.7 c  (0.7) | 10.3 d  (3.0)  7.7 d  (4.7)  26.4 d  (0.9)  21.2 d  (22.1)  0.5 bd  (0.6) |  | 9.6 b  (2.8)  11.1 a  (4.2)  27.0 e  (1.2)  27.6 e  (26.3)  1.1 a  (0.8) | 9.6 b  (2.8)  11.1 a  (4.2)  26.8 f  (1.0)  26.1 e  (24.8)  0.5 d  (0.5) | 9.7 b  (2.8)  11.1 a  (4.2)  26.8 f  (1.0)  26.2 e  (25.0)  0.5 d  (0.5) | 9.6 b  (2.8)  11.0 a  (4.2)  26.7 f  (0.9)  26.6 e  (24.7)  0.4 d  (0.5) |

**Table S5.** Means of *Myotis grisescens* pulse parameters selected from the main library by different AnalookW filters and means of a consensus set of pulses selected by all four filters. Standard deviations given in parentheses. Parameters explained in text. Different letters indicate significantly different means (α = 0.05) according to pairwise Wilcoxon rank sum tests.

|  |  | All Selected | | | |  | Consensus | | | |
| --- | --- | --- | --- | --- | --- | --- | --- | --- | --- | --- |
| Parameter | Units | BM  (n = 3,213) | BCID  (n = 2,823) | WEST 1  (n = 1,562) | WEST 2  (n = 1,320) |  | BM  (n = 1,212) | BCID  (n = 1,212) | WEST 1  (n = 1,212) | WEST 2  (n = 1,212) |
| Dur  Sweep  Fc  Sc  Tail | Ms  kHz  kHz  octaves/s  ms | 4.0 a  (1.7)  18.6 a  (9.6)  47.9 a  (6.2)  97.6 a  (1123.5)  0.8 a  (0.6) | 4.4 b  (1.4)  18.4 a  (10.1)  46.6 b  (1.7)  60.6 b  (29.7)  0.3 b  (0.4) | 4.5 bc  (1.5)  19.5 b  (9.4)  46.4 bc  (1.6)  66.2 c  (36.1)  0.3 bc  (0.4) | 4.5 bc  (1.4)  19.4 b  (8.9)  46.4 bc  (1.3)  61.7 c  (24.5)  0.3 c  (0.4) |  | 4.6 c  (1.4)  20.0 b  (8.8)  47.4 d  (1.5)  62.3 c  (2.3)  0.8 a  (0.6) | 4.6 c  (1.4)  20.0 b  (8.8)  46.4 bc  (1.3)  60.8 bc  (24.2)  0.3 bc  (0.3) | 4.6 c  (1.4)  20.0 b  (8.8)  46.3 c  (1.3)  62.4 c  (2.5)  0.3 bc  (0.3) | 4.6 c  (1.4)  19.8 b  (8.6)  46.4 bc  (1.3)  61.4 c  (24.0)  0.3 bc  (0.4) |

**Table S6.** Means of *Myotis leibii* pulse parameters selected from the main library by different AnalookW filters and means of a consensus set of pulses selected by all four filters. Standard deviations given in parentheses. Parameters explained in text. Different letters indicate significantly different means (α = 0.05) according to pairwise Wilcoxon rank sum tests.

|  |  | All Selected | | | |  | Consensus | | | |
| --- | --- | --- | --- | --- | --- | --- | --- | --- | --- | --- |
| Parameter | Units | BM  (n = 385) | BCID  (n = 154) | WEST 1  (n = 203) | WEST 2  (n = 75) |  | BM  (n = 62) | BCID  (n = 62) | WEST 1  (n = 62) | WEST 2  (n = 62) |
| Dur  Sweep  Fc  Sc  Tail | Ms  kHz  kHz  octaves/s  ms | 2.2 a  (0.9)  26.0 a  (13.4)  49.7 a  (6.4)  227.9 a  (118.4)  0.5 a  (0.3) | 3.0 b  (0.6)  34.0 b  (12.1)  48.1 b  (3.1)  170.3 b  (72.8)  0.4 b  (0.3) | 2.2 a  (0.9)  25.4 a  (10.9)  47.9 b  (2.6)  203.8 ac  (74.9)  0.2 c  (0.3) | 2.9 b  (0.6)  32.2 b  (9.5)  48.8 b  (3.3)  190.2 b  (78.1)  0.5 ab  (0.3) |  | 3.0 b  (0.6)  33.8 b  (9.3)  47.9 b  (2.4)  157.7 b  (59.9)  0.5 b  (0.2) | 3.0 b  (0.6)  33.7 b  (9.3)  47.5 bc  (2.7)  165.9 b  (55.2)  0.4 bd  (0.3) | 3.0 b  (0.6)  33.3 b  (9.0)  46.5 c  (1.9)  155.8 b  (42.8)  0.3 cd  (0.3) | 3.0 b  (0.6)  33.2 b  (9.0)  48.1 b  (2.9)  178.2 bc  (64.1)  0.5 ab  (0.3) |

**Table S7.** Means of *Myotis lucifugus* pulse parameters selected from the main library by different AnalookW filters and means of a consensus set of pulses selected by all four filters. Standard deviations given in parentheses. Parameters explained in text. Different letters indicate significantly different means (α = 0.05) according to pairwise Wilcoxon rank sum tests.

|  |  | All Selected | | | |  | Consensus | | | |
| --- | --- | --- | --- | --- | --- | --- | --- | --- | --- | --- |
| Parameter | Units | BM  (n = 7,246) | BCID  (n = 5,640) | WEST 1  (n = 3,146) | WEST 2  (n = 2,346) |  | BM  (n = 2,222) | BCID  (n = 2,222) | WEST 1  (n = 2,222) | WEST 2  (n = 2,222) |
| Dur  Sweep  Fc  Sc  Tail | Ms  kHz  kHz  octaves/s  ms | 3.8 a  (1.6)  19.1 a  (9.6)  43.1 a  (6.4)  121.9 a  (136.4)  0.8 a  (0.7) | 4.3 b  (1.3)  19.8 b  (9.6)  41.1 b  (2.5)  93.2 bc  (34.2)  0.4 b  (0.5) | 4.4 c  (1.4)  20.7 c  (8.7)  40.9 b  (2.2)  99.9 d  (49.0)  0.3 bc  (0.4) | 4.5 cd  (1.3)  20.4 c  (8.4)  41.1 c  (2.0)  94.3 cd  (30.9)  0.4 b  (0.5) |  | 4.6 d  (1.3)  20.9 c  (8.3)  42.3 d  (2.9)  90.9 b  (29)  0.8 a  (0.7) | 4.6 d  (1.2)  20.8 c  (8.2)  40.9 b  (2.1)  91.4 b  (29.6)  0.3 d  (0.4) | 4.6 d  (1.3)  20.9 c  (8.3)  40.8 b  (1.9)  93.4 bc  (3.1)  0.3 c  (0.4) | 4.6 d  (1.2)  20.6 c  (8.2)  41.1 c  (2.0)  93.8 cd  (29.5)  0.4 b  (0.5) |

**Table S8.** Means of *Myotis septentrionalis* pulse parameters selected from the main library by different AnalookW filters and means of a consensus set of pulses selected by all four filters. Standard deviations given in parentheses. Parameters explained in text. Different letters indicate significantly different means (α = 0.05) according to pairwise Wilcoxon rank sum tests.

|  |  | All Selected | | | |  | Consensus | | | |
| --- | --- | --- | --- | --- | --- | --- | --- | --- | --- | --- |
| Parameter | Units | BM  (n = 3,426) | BCID  (n = 1,220) | WEST 1  (n = 1,615) | WEST 2  (n = 623) |  | BM  (n = 532) | BCID  (n = 532) | WEST 1  (n = 532) | WEST 2  (n = 532) |
| Dur  Sweep  Fc  Sc  Tail | Ms  kHz  kHz  octaves/s  ms | 2.1 a  (0.8)  27.5 a  (15.4)  46.9 a  (8.1)  277.5 a  (188.2)  0.6 a  (0.3) | 2.9 b  (0.5)  37.4 b  (12.6)  45.8 b  (6.5)  253.1 b  (65.0)  0.7 b  (0.4) | 2.3 c  (0.8)  27.2 c  (11.8)  43.8 c  (3.2)  253.8 b  (76.3)  0.3 c  (0.3) | 2.8 d  (0.6)  33.2 d  (10.8)  45.7 d  (3.8)  256.6 ab  (57.5)  0.7 d  (0.2) |  | 3.0 b  (0.5)  35.4 e  (10.8)  45.8 bd  (4.9)  234.6 c  (55.5)  0.7 b  (0.4) | 2.9 b  (0.5)  35.1 e  (10.6)  45.6 bd  (4.3)  250.7 b  (52.8)  0.7 e  (0.3) | 3.0 b  (0.5)  35.2 e  (10.6)  42.9 e  (2.5)  222.8 d  (108.4)  0.4 f  (0.3) | 2.9 b  (0.5)  34.7 de  (10.3)  45.6 bd  (3.8)  254.0 b  (57.3)  0.7 de  (0.2) |

**Table S9.** Means of *Nycticeius humeralis* pulse parameters selected from the main library by different AnalookW filters and means of a consensus set of pulses selected by all four filters. Standard deviations given in parentheses. Parameters explained in text. Different letters indicate significantly different means (α = 0.05) according to pairwise Wilcoxon rank sum tests.

|  |  | All Selected | | | |  | Consensus | | | |
| --- | --- | --- | --- | --- | --- | --- | --- | --- | --- | --- |
| Parameter | Units | BM  (n = 3,871) | BCID  (n = 3,669) | WEST 1  (n = 1,607) | WEST 2  (n = 971) |  | BM  (n = 812) | BCID  (n = 812) | WEST 1  (n = 812) | WEST 2  (n = 812) |
| Dur  Sweep  Fc  Sc  Tail | Ms  kHz  kHz  octaves/s  ms | 6.1 a  (3.1)  16.5 a  (8.4)  36.0 a  (8.4)  99.1 a  (230.3)  1.2 a  (1.0) | 6.7 b  (2.6)  14.4 b  (8.6)  36.8 bc  (1.8)  32.9 b  (31.1)  0.5 b c  (0.5) | 7.2 c  (2.8)  16.1 acd  (8.2)  36.6 d  (1.9)  37.0 b  (48.7)  0.5 b  (0.5) | 7.3 c  (2.8)  15.2 c  (7.8)  36.6 d  (1.7)  34.8 b  (34.8)  0.5 b  (0.5) |  | 7.3 c  (2.8)  16.8 d  (7.4)  36.9 b  (1.8)  38.7 c  (3.6)  1.2 a  (0.9) | 7.2 c  (2.7)  16.6 ad  (7.3)  36.7 cd  (1.6)  35.2 b  (3.7)  0.5 b  (0.5) | 7.3 c  (2.8)  16.8 d  (7.3)  36.7 d  (1.6)  36.1 b  (3.8)  0.5 b  (0.5) | 7.2 c  (2.7)  16.5 ad  (7.3)  36.6 cd  (1.6)  36.6 bc  (36.0)  0.5 b  (0.5) |

**Table S10.** Means of *Perimyotis subflavus* pulse parameters selected from the main library by different AnalookW filters and means of a consensus set of pulses selected by all four filters. Standard deviations given in parentheses. Parameters explained in text. Different letters indicate significantly different means (α = 0.05) according to pairwise Wilcoxon rank sum tests.

|  |  | All Selected | | | |  | Consensus | | | |
| --- | --- | --- | --- | --- | --- | --- | --- | --- | --- | --- |
| Parameter | Units | BM  (n = 6,158) | BCID  (n = 6,550) | WEST 1  (n = 3,842) | WEST 2  (n = 2,698) |  | BM  (n = 2,156) | BCID  (n = 2,156) | WEST 1  (n = 2,156) | WEST 2  (n = 2,156) |
| Dur  Sweep  Fc  Sc  Tail | Ms  kHz  kHz  octaves/s  ms | 5.0 a  (1.9)  15.0 a  (8.9)  42.1 a  (7.1)  68.1 a  (187.9)  0.9 a  (0.7) | 5.3 b  (1.5)  12.1 b  (8.5)  42.8 b  (1.3)  24.6 b  (23.6)  0.3 b  (0.4) | 5.5 c  (1.5)  11.5 b  (6.9)  42.7 b  (1.4)  24.8 c  (33.3)  0.3 c  (0.4) | 5.4 bd  (1.4)  11.1 b  (5.8)  42.8 bc  (1.3)  25.2 d  (17.9)  0.3 d  (0.4) |  | 5.5 c  (1.4)  12.6 c  (5.8)  43.0 a  (1.3)  27.6 e  (18.1)  0.7 e  (0.5) | 5.5 c  (1.4)  12.5 c  (5.8)  42.9 c  (1.2)  23.8 f  (1.8)  0.3 d  (0.3) | 5.5 c  (1.4)  12.5 c  (5.6)  42.9 c  (1.2)  24.4 f  (1.8)  0.3 d  (0.3) | 5.5 cd  (1.4)  12.2 c  (5.5)  42.8 c  (1.2)  25.6 d  (1.8)  0.3 d  (0.4) |
